# Supplementary figures and images for: A tRNA processing enzyme is a key regulator of the mitochondrial unfolded protein response
Source: eLife. 2022 Apr 22;11:e71634. doi: 10.7554/eLife.71634 (PMC9064297; doi:10.7554/eLife.71634)

**Figure 5 – source data 1**

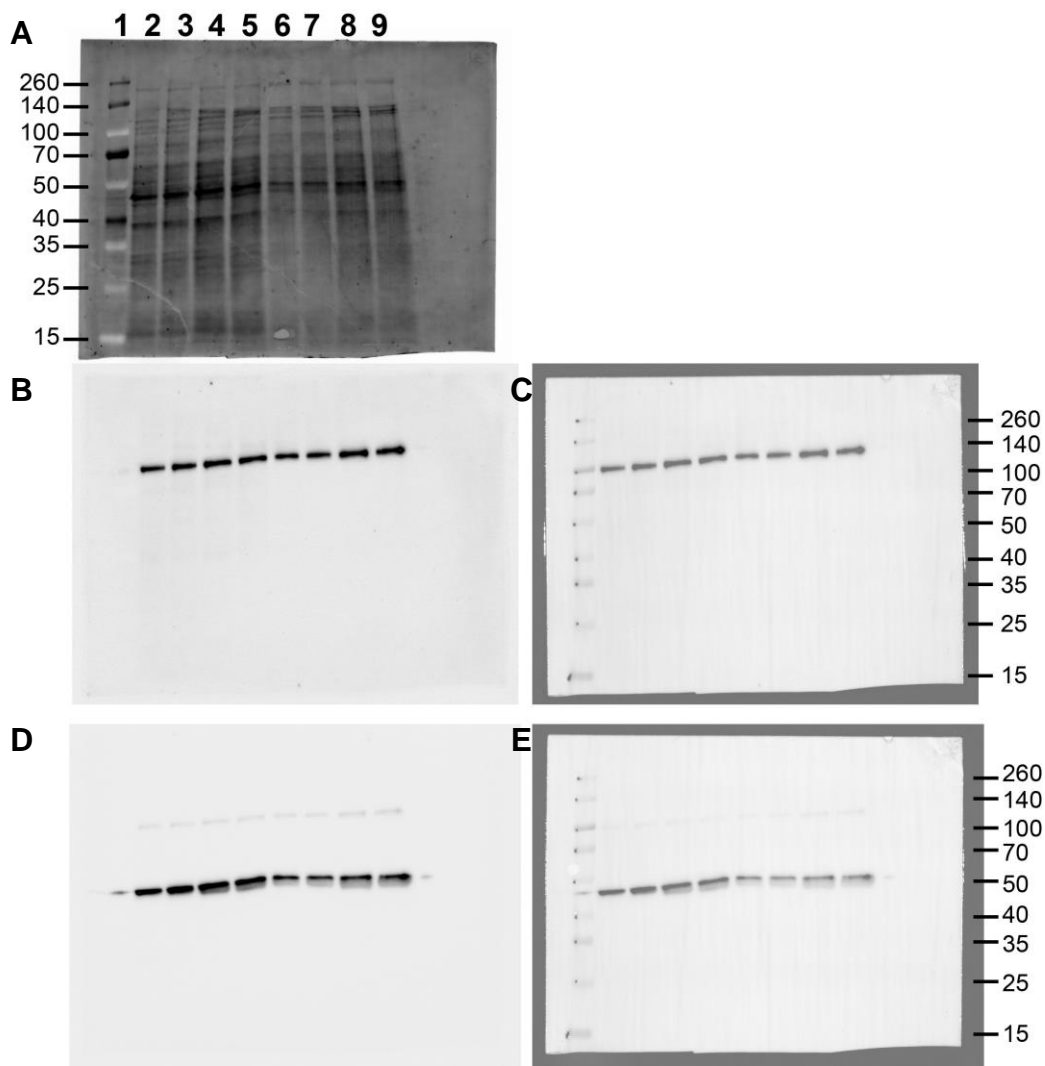

Supplement: Figure 5—source data 1. — All panels are the same membrane. (A) Image of stain-free blot for total protein from day 1 adult wildtype and hoe-1(ΔNES) animals. Four biological replicates of each condition: Lane #1 BR Spectra Protein Ladder – ladder bands in kDa denoted, Lane #2–5 wildtype and #6–9 hoe-1(ΔNES). (B) Chemiluminescence image of blot for DVE-1::GFP using GFP primary antibody. (C) Composite image of chemiluminescence and colorimetric images of blot for DVE-1::GFP to show bands relative to ladder. (D) Chemiluminescence image of blot for actin using β-actin primary antibody. (E) Composite image of chemiluminescence and colorimetric images of blot for actin to show bands relative to ladder. [file elife-71634-fig5-data1.zip › Figure 5 source data 1.pdf]

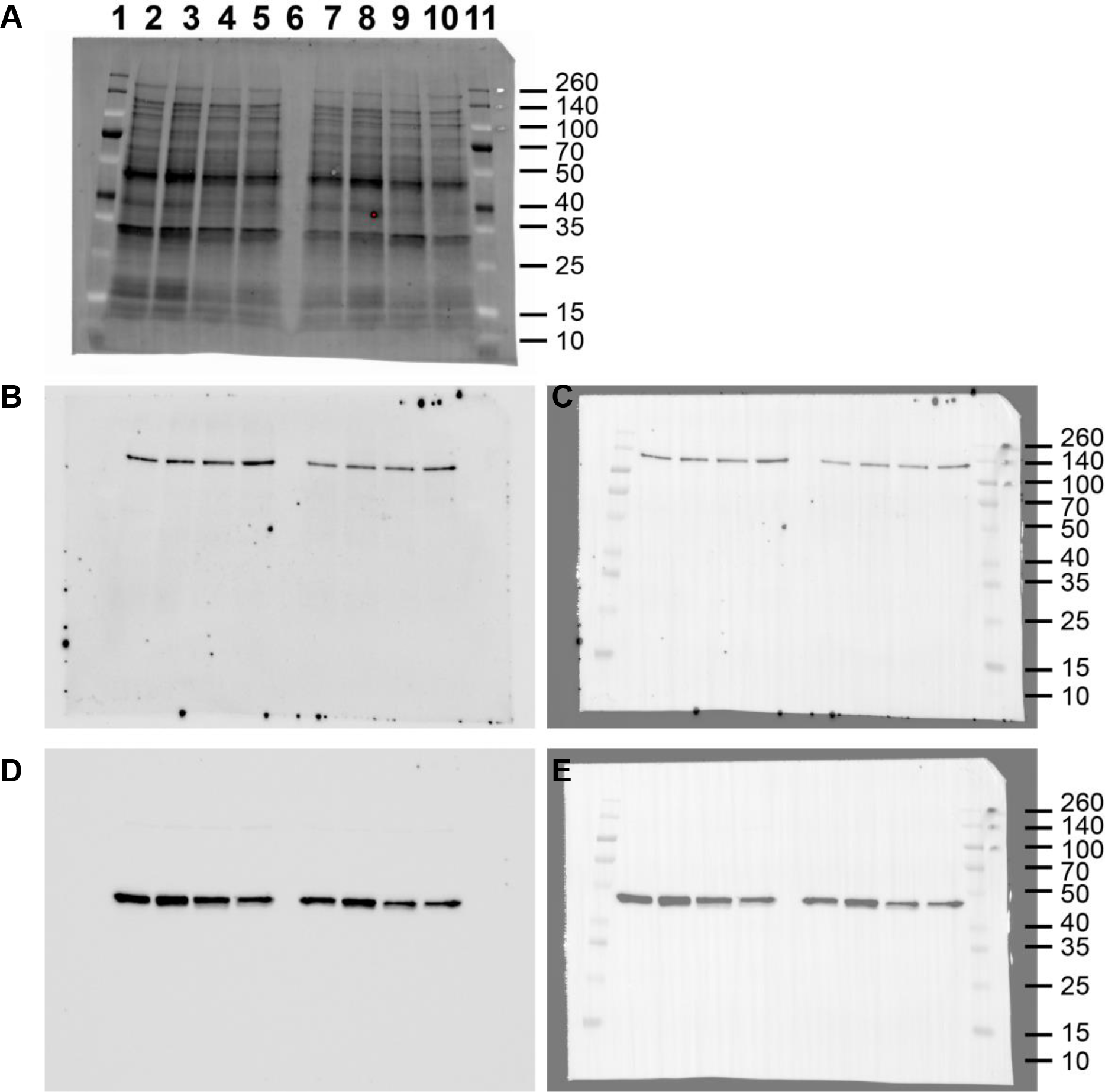

Supplement: Figure 8—source data 1. — All panels are the same membrane. (A) Image of stain-free blot for total protein from day 1 adult wildtype and nuo-6(qm200) animals on control and atfs-1 RNAi. Two biological replicates of each condition: Lane # 1&11 BR Spectra Protein Ladder – ladder bands in kDa denoted. Lane # 2&7 wildtype on control RNAi, 3&8 wildtype on atfs-1 RNAi, 4&9 nuo-6(qm200) on control RNAi, and 5&10 nuo-6(qm200) on atfs-1 RNAi. Lane # 6 empty. (B) Chemiluminescence image of blot for HOE-1::GFP using GFP primary antibody. (C) Composite image of chemiluminescence and colorimetric images of blot for HOE-1::GFP to show bands relative to ladder. (D) Chemiluminescence image of blot for actin using β-actin primary antibody. (E) Composite image of chemiluminescence and colorimetric images of blot for actin to show bands relative to ladder. [file elife-71634-fig8-data1.zip › Figure 8 source data 1.tif]

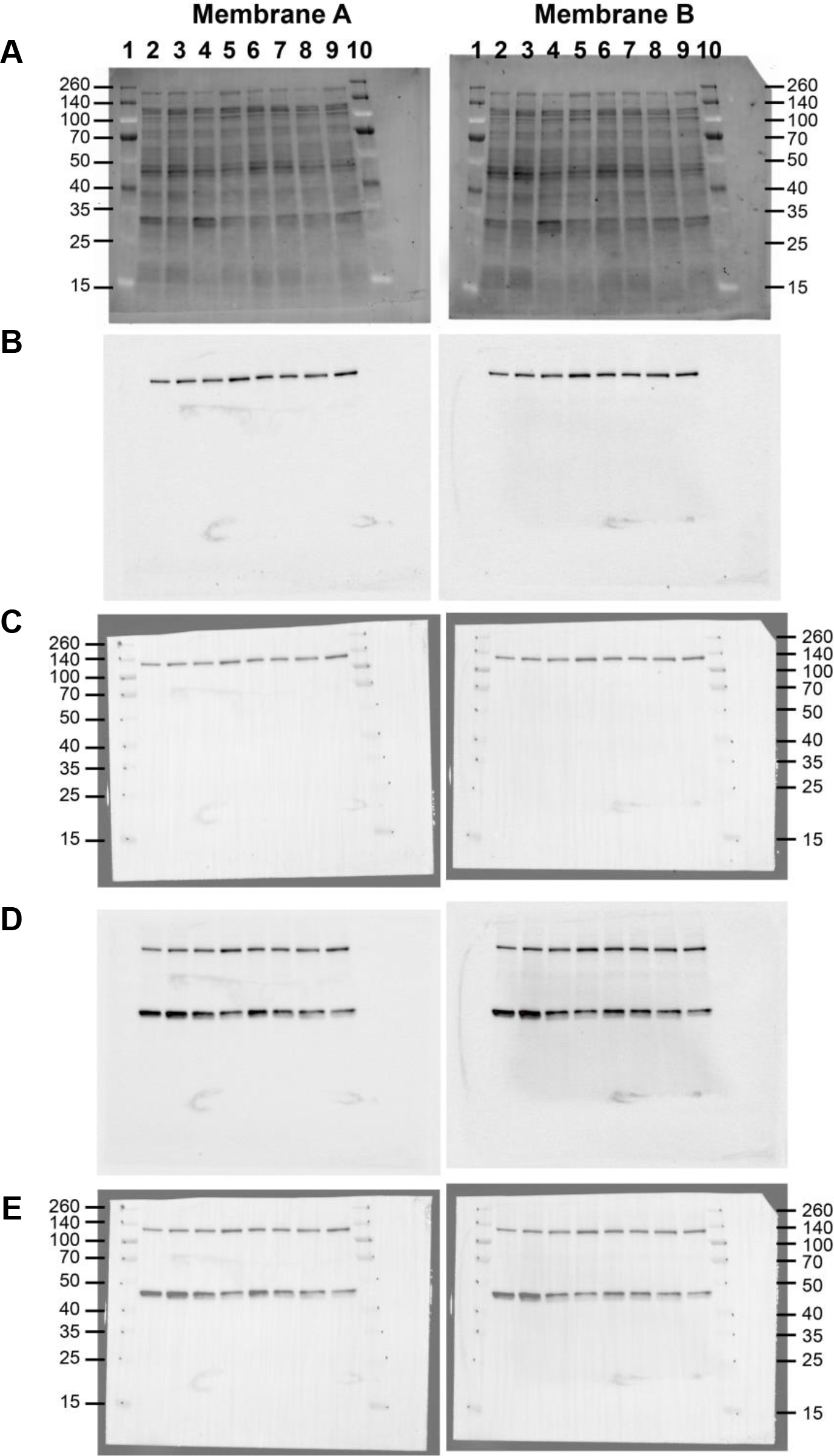

Supplement: Figure 8—source data 2. — Samples were loaded and ran on two separate membranes simultaneously (Membrane A and Membrane B). All panels in each column are the same membrane. (A) Image of stain-free blots for total protein from day 1 adult wildtype and nuo-6(qm200) animals on control and atfs-1 RNAi. Two biological replicates on each blot of each condition: Lane # 1&10 BR Spectra Protein Ladder – ladder bands in kDa denoted. Lane # 2&6 wildtype on control RNAi, 3&7 wildtype on atfs-1 RNAi, 4&8 nuo-6(qm200) on control RNAi, and 5&9 nuo-6(qm200) on atfs-1 RNAi. (B) Chemiluminescence image of blots for HOE-1::GFP using GFP primary antibody. (C) Composite images of chemiluminescence and colorimetric images of blots for HOE-1::GFP to show bands relative to ladder. (D) Chemiluminescence images of blots for actin using β-actin primary antibody. (E) Composite images of chemiluminescence and colorimetric images of blots for actin to show bands relative to ladder. [file elife-71634-fig8-data2.zip › Figure 8 source data 2.tif]

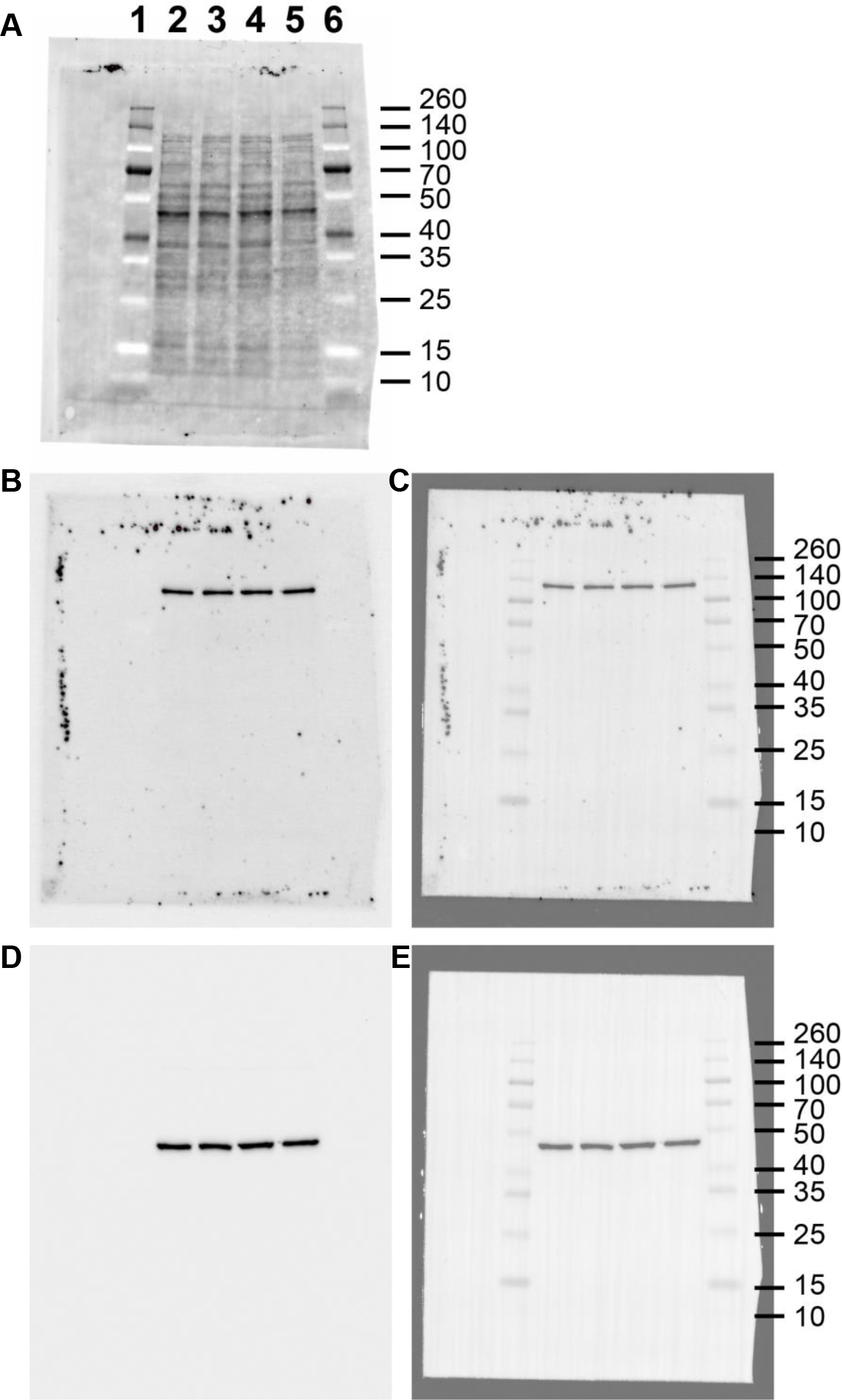

Supplement: Figure 8—source data 3. — All panels are the same membrane. (A) Image of stain-free blot for total protein from day 1 adult wildtype and atfs-1(et15) animals. Two biological replicates of each condition: Lane # 1&6 BR Spectra Protein Ladder – ladder bands in kDa denoted. Lane #2&4 wildtype and #3&5 atfs-1(et15). (B) Chemiluminescence image of blot for HOE-1::GFP using GFP primary antibody. (C) Composite image of chemiluminescence and colorimetric images of blot for HOE-1::GFP to show bands relative to ladder. (D) Chemiluminescence image of blot for actin using β-actin primary antibody. (E) Composite image of chemiluminescence and colorimetric images of blot for actin to show bands relative to ladder. [file elife-71634-fig8-data3.zip › Figure 8 source data 3.tif]

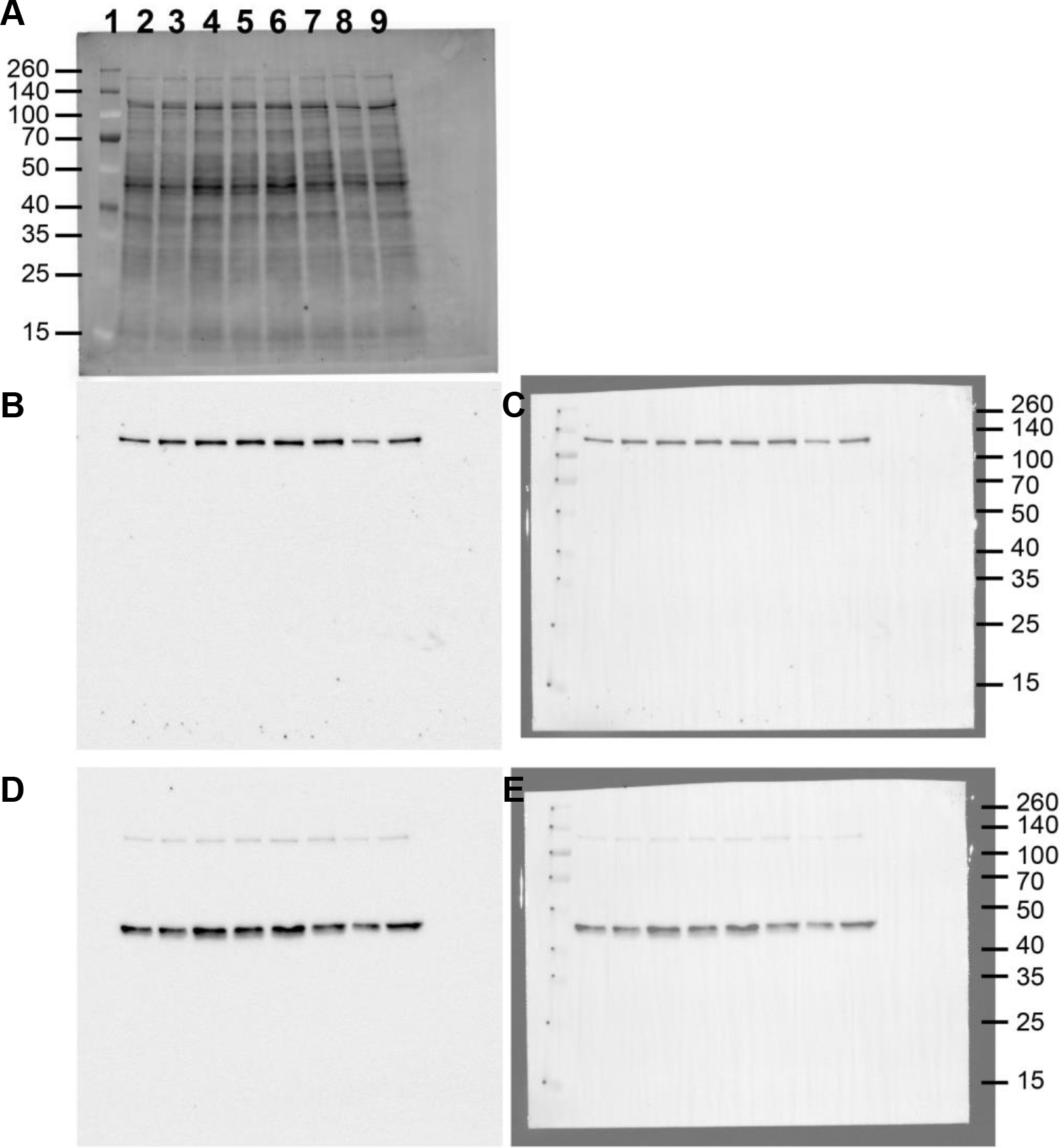

Supplement: Figure 8—source data 4. — All panels are the same membrane. (A), Image of stain-free blot for total protein from day 1 adult wildtype and atfs-1(et15) animals. Four biological replicates of each condition: Lane # 1 BR Spectra Protein Ladder – ladder bands in kDa denoted. Lanes #2,4,6,8 wildtype and #3,5,7,9 atfs-1(et15). (B) Chemiluminescence image of blot for HOE-1::GFP using GFP primary antibody. (C) Composite image of chemiluminescence and colorimetric images of blot for HOE-1::GFP to show bands relative to ladder. (D) Chemiluminescence image of blot for actin using β-actin primary antibody. (E) Composite image of chemiluminescence and colorimetric images of blot for actin to show bands relative to ladder. [file elife-71634-fig8-data4.zip › Figure 8 source data 4.tif]
